# TMEM16A ameliorates vascular remodeling by suppressing autophagy via inhibiting Bcl-2-p62 complex formation

**DOI:** 10.7150/thno.41028

**Published:** 2020-03-04

**Authors:** Xiao-Fei Lv, Ya-Juan Zhang, Xiu Liu, Hua-Qing Zheng, Can-Zhao Liu, Xue-Lin Zeng, Xiang-Yu Li, Xiao-Chun Lin, Cai-Xia Lin, Ming-Ming Ma, Fei-Ran Zhang, Jin-Yan Shang, Jia-Guo Zhou, Si-Jia Liang, Yong-Yuan Guan

**Affiliations:** Department of Pharmacology and Cardiac & Cerebral Vascular Research Center, Zhongshan School of Medicine, Sun Yat-Sen University, Guangzhou, China.

**Keywords:** transmembrane member 16A, autophagy, vascular remodeling, vacuolar protein sorting 34, smooth muscle cell

## Abstract

**Rationale**: Transmembrane member 16A (TMEM16A) is a component of calcium-activated chloride channels that regulate vascular smooth muscle cell (SMC) proliferation and remodeling. Autophagy, a highly conserved cellular catabolic process in eukaryotes, exerts important physiological functions in vascular SMCs. In the current study, we investigated the relationship between TMEM16A and autophagy during vascular remodeling.

**Methods**: We generated a transgenic mouse that overexpresses TMEM16A specifically in vascular SMCs to verify the role of TMEM16A in vascular remodeling. Techniques employed included immunofluorescence, electron microscopy, co-immunoprecipitation, and Western blotting.

**Results**: Autophagy was activated in aortas from angiotensin II (AngII)-induced hypertensive mice with decreased TMEM16A expression. The numbers of light chain 3B (LC3B)-positive puncta in aortas correlated with the medial cross-sectional aorta areas and TMEM16A expression during hypertension. SMC-specific TMEM16A overexpression markedly inhibited AngII-induced autophagy in mouse aortas. Moreover, in mouse aortic SMCs (MASMCs), AngII-induced autophagosome formation and autophagic flux were blocked by TMEM16A upregulation and were promoted by TMEM16A knockdown. The effect of TMEM16A on autophagy was independent of the mTOR pathway, but was associated with reduced kinase activity of the vacuolar protein sorting 34 (VPS34) enzyme. Overexpression of VPS34 attenuated the effect of TMEM16A overexpression on MASMC proliferation, while the effect of TMEM16A downregulation was abrogated by a VPS34 inhibitor. Further, co-immunoprecipitation assays revealed that TMEM16A interacts with p62. TMEM16A overexpression inhibited AngII-induced p62-Bcl-2 binding and enhanced Bcl-2-Beclin-1 interactions, leading to suppression of Beclin-1/VPS34 complex formation. However, TMEM16A downregulation showed the opposite effects.

**Conclusion**: TMEM16A regulates the four-way interaction between p62, Bcl-2, Beclin-1, and VPS34, and coordinately prevents vascular autophagy and remodeling

## Introduction

Vascular smooth muscle cell (VSMC) proliferation is considered an important feature of hypertension-induced vascular remodeling 1-3. Co-ordination of VSMC proliferation, migration, and extracellular matrix accumulation increases the ratio of wall to lumen diameter in arteries, and leads to vascular remodeling 4,5. Angiotensin II (AngII), a bioactive peptide of the renin-angiotensin-aldosterone system, is critical in vascular remodeling during hypertension 6,7. Increased AngII level may be associated with the development of multiple cardiovascular diseases, such as hypertension, stroke, atherosclerosis, and restenosis 6-8. Increasing evidence has demonstrated that elevated VSMC proliferation is the major mechanism underlying AngII-induced cardiovascular remodeling 2,3,6. Although VSMC proliferation is an important contributor to intimal hyperplasia, the mechanisms whereby AngII triggers this process remain largely unclear.

Autophagy is a highly conserved cellular catabolic process that contributes intracellular homeostasis by recycling or degrading proteins and damaged organelles in lysosomal compartments 9. In the vasculature, abnormal autophagy has been implicated in various cardiovascular diseases, including hypertension, heart failure, atherosclerosis, and restenosis 4,10-14. Importantly, previous evidence demonstrated that autophagy critically regulated VSMC proliferation and survival, suggesting that modulating autophagy activity may be a viable therapeutic strategy for vascular remodeling treatment 1,9,15. Moreover, AngII has been shown to induce autophagy in VSMCs and endothelial cells; which may be associated with AngII-induced cell injury 7,8. However, the specific relationship between autophagy and AngII-induced vascular remodeling is unknown. Moreover, the precise role of autophagy in VSMC proliferation remains controversial because of inconsistent or conflicting data 1,15. Therefore, it is a challenge to understand how autophagy regulates VSMC function in response to specific stimuli or pathological conditions.

Recently, chloride channels have become appreciated as important regulators of autophagy 16. Several chloride channels help regulate autophagy, such as CFTR, ClC-3, ClC-7, and LRRC8 17-20. Transmembrane member 16A (TMEM16A) is a subunit of calcium-activated chloride channels (CaCCs) with important physiological functions in VSMCs and other cell types 2, 3, 20-23. Previous findings from our group and other groups have revealed that TMEM16A is associated with the regulation of endothelial function, vascular remodeling, vasoconstriction, ventricular hypertrophy, and blood pressure 3, 20, 22, 23. Moreover, we also provided evidence that TMEM16A expression was decreased in smooth muscle cells (SMCs) from the basilar arteries of hypertensive rats and that this decrease promoted cell proliferation and hypertension-induced cerebrovascular remodeling 2. However, whether and how TMEM16A participates in autophagy-mediated vascular remodeling remains enigmatic.

In this study, we investigated the contributions of autophagy to AngII-induced vascular SMC proliferation and remodeling, and examined whether TMEM16A is involved in these processes. Our results demonstrate that TMEM16A prevents vascular SMC proliferation and remodeling by inhibiting SMC autophagy.

## Materials and methods

### Materials and reagents

Dulbecco's modified Eagle's medium (DMEM), medium 199 (M199), collagenase type-III, elastase, soybean trypsin inhibitor, fetal bovine serum (FBS), penicillin, streptomycin, heparin, endothelial cell growth factor (ECGF), and the Lipofectamine 2000 reagent were from Invitrogen (NY, USA). 5-bromo-2'-deoxyuridine (BrdU), an anti-BrdU antibody, and AngII were from Sigma-Aldrich (MO, USA). Rapamycin, 3-MA, and SAR405 were from Selleck Chemical (TX, USA).

### SMC-specific TMEM16A transgenic mice

All animal experiments were performed according to the policies of the Sun Yat-Sen University Committee for Animal Research and conformed to the “Guide for the Care and Use of Laboratory Animals” of the National Institute of Health in China. TMEM16A transgenic mice were designed and produced as we previously described 20, 24. Briefly, the transgene construct, (pRP.ExBi-CMV-LoxP-Stop-LoxP-TMEM16A) was microinjected into fertilized mouse embryos to generate transgenic mice. TMEM16A was not overexpressed in this strain because of the stop codon system. TMEM16A SMC-specific transgenic mice (TM^SMC Tg^) were generated by crossing transgenic founders with mice expressing Cre from a SM22α promoter (SM22α-Cre). Mice were genotyped by PCR using the following primers: Loxp site: forward 5′-TCATGTCTGGATCCCCATCAAGC-3′ and reverse 5′-GAGTACTTCTCGGGGACCCTCA-3′; Cre forward: 5′-GCGGTCTGGCAGTAAAAACTATC-3′ and reverse 5′-GTGAAACAGCATTGCTGTCACTT-3′. Transgenic mice that did not express SM22α-Cre were referred to as TM^con^ mice.

### Animal model

Male C57BL/6J mice were obtained from the Experimental Animal Center of Sun Yat-Sen University. An AngII-induced hypertension model was established as previously described 3, 20. Briefly, wild-type, TM^con^, or TM^SMC Tg^ mice were anesthetized with 2% pentobarbital sodium and implanted subcutaneously with osmotic minipumps (Alzet®, Cupertino; model 1002, CA, USA) for AngII delivery (1.5 mg•kg^-1^•d^-1^) for 4 weeks. Sham-operated mice were implanted with osmotic minipumps containing 0.9% saline. The systolic blood pressure (SBP) of the mice was measured weekly by non-invasive tail-cuff plethysmography (BP-98A, Softron Co, Tokyo, Japan).

### Cell isolation and culture

Mouse aortic SMCs (MASMCs) and mouse aortic endothelial cells (MAECs) were isolated and cultured as described previously 20. Briefly, aortas were carefully dissected and cleaned of connective tissue; digested for 30 min at 37 ºC in DMEM containing 200 U/mL type-III collagenase, 0.1 mg/mL elastase, and 0.5 mg/mL soybean trypsin inhibitor; and incubated for another 45 min 37 ºC in medium containing 130 U/mL type-III collagenase, 0.1 mg/mL elastase, and 0.5 mg/mL soybean trypsin inhibitor. The media tissue was minced with scissors and digested further at 37 ºC for 60 min, and then MASMCs were harvested by centrifugation at 200 × *g* for 3 min and cultured in DMEM containing 20% FBS, 100 U/mL penicillin, and 100 U/mL streptomycin. To isolate MAECs, the aorta was first opened longitudinally and cut into small pieces. The explants were placed intima side down in a fibronectin-coated culture dish and cultured in M199 medium containing 20% FBS, 25 U/mL heparin, 10 ng/mL ECGF, 100 U/mL penicillin, and 100 U/mL streptomycin at 37 ºC in 5% CO_2_. Approximately 5 days later, the cells began migrating from the aortic segments.

### Adenoviral infection

An adenovirus encoding monomeric red fluorescent protein (mRFP), green fluorescent protein (GFP), and light chain 3 (LC3) in a single open reading frame (tandem mRFP-GFP-LC3 adenovirus) was constructed by HanBio Technology (Shanghai, China). The human TMEM16A adenovirus was purchased from Sunbio Biotechnology (Shanghai, China). Adenoviral infection of MASMCs was performed in serum- and antibiotic-free DMEM for 6 h. Subsequently, the cells were transferred to fresh medium containing serum and antibiotics for another 42 h. The Lacz adenovirus (Sunbio Biotechnology) was used as a negative control.

### Small interfering RNA (siRNA) transfection

siRNA duplexes against mouse TMEM16A mRNA (5′-CUGCUCAAGUUUGUGAACUTT-3′) and a scrambled siRNA were designed and synthesized by Qiagen (CA, USA). MASMCs were transfected with TMEM16A or scrambled siRNA for 48 h, using HiPerfect Transfection Reagent (Qiagen), according to the manufacturer's instructions.

### Plasmid transfection

TMEM16A cDNA was kindly provided by Dr. LY Jan (University of California, CA, USA), after which it was epitope-tagged with DNA coding for mRFP and HA, and subcloned into pMSCV using the overlap-extension PCR-cloning method. The His-p62 plasmid was a kind gift from Dr. Jian Pan (Sun Yat-Sen University, Guangzhou, China). Vacuolar protein sorting 34 (VPS34) plasmid was obtained from Addgene (MA, USA). Plasmids were transfected using Lipofectamine 2000, according to the manufacturer's instructions.

### Western blotting

Western blotting was performed as previously described 2,25. Briefly, aliquots of each sample containing 40 µg of protein were separated by sodium dodecyl sulfate-polyacrylamide gel electrophoresis and then transferred to polyvinylidene fluoride membranes (Millipore, MA, USA). After blocking with non-fat dry milk for 1 h at room temperature, the membranes were probed overnight at 4 ºC with primary antibodies against the following proteins: TMEM16A (ab53212; 1:1,000), VPS34 (ab124905; 1:500) obtained from Abcam, MA, USA; light chain 3B (LC3B)-I/II (#3868, 1:1,000), p62 (#39749, 1:1,000), p-AKT (Ser473; #4060; 1:1,000), AKT (#4691; 1:1,000), p-mTOR (Ser2448; #5536; 1:1,000), mTOR (#2983; 1:1,000), p-p70S6K (Ser371 #9208; 1:500), p70S6K (#2708; 1:500) from Cell Signaling Technology (MA, USA); Beclin-1 (sc-48341; 1:1,000), p-4EBP1 (sc-9977, 1:500), and 4EBP1 (Ser65; sc-293124; 1:500) from Santa Cruz Biotechnology (CA, USA); and Bcl-2 (BM4985; 1:1,000) and β-actin (M01263-2; 1:1,000) from Boster Biological Technology (Wuhan, China). Next, the membranes were incubated with HRP-linked anti-mouse IgG (#7076; 1:1,000) or HRP-linked anti-rabbit IgG (#7074; 1:1,000) secondary antibodies (Cell Signaling Technology), and the blots were visualized using the Immobilon Western Chemiluminescent HRP Substrate Kit (Millipore). Target band densities were measured using the ImageJ program (NIH, Maryland, USA).

### Immunofluorescence

The thoracic aortas were isolated and embedded in optimal cutting temperature compound (Sakura, Japan) for sectioning at an 8-um thickness. Frozen slides were incubated overnight at 4ºC with antibodies against LC3B (NB100-2220; 1:100; Novus Biologicals, CO, USA) and alpha-smooth muscle actin (α-SMA) (BM0002; 1:100; Boster Biological Technology) and then treated with FITC-labeled anti-rabbit IgG (31635; 1:100) and Cy3-labeled anti-mouse IgG (A10521; 1:100) secondary antibodies (Invitrogen, CA, USA). Nuclei were counterstained with 4′,6-diamidino-2-phenylindole (DAPI). MASMCs were infected with the mRFP-GFP-LC3 adenovirus for 48 h. The puncta in thoracic aortas and MASMCs were viewed under a confocal microscope (Zeiss LSM800, Carl Zeiss, Munich, Germany) with z-stacks and 63× objective lens. The number of puncta was analyzed using the ImageJ program. For endogenous colocalization studies, MASMCs were incubated with TMEM16A (ab53212; 1:50), VPS34 (ab124905; 1:50) (Abcam), p62 (sc-48402; 1:100), Beclin-1 (sc-48341; 1:1000) (Santa Cruz Biotechnology), and Bcl-2 (BM4985; 1:100) (Boster Biological Technology) antibodies, and processed for colocalization observation with a confocal microscope.

### Histologic analyses

For immunohistochemical staining of Ki67, thoracic aortas sections were incubated with Ki67 antibody (ab15580; 1:100; Abcam) overnight at 4 °C and then stained with biotinylated secondary anti-rabbit antibody at room temperature for 1 h, followed by visualization with 3,3-diaminobenzidine tetrachloride and counterstaining with hematoxylin. The sections were also stained with hematoxylin-eosin (H&E) for histopathological examination. All images were acquired using a light microscope (BX51WI, Olympus, Tokyo, Japan). The wall diameter (WD) and lumen diameter (LD) were measured using ImageJ software independently by three experienced histopathologists who were blinded to group assignment. The media thickness was then calculated. The mean value of the medial cross-sectional area (CSA) was obtained using the following equation: CSA = (π/4) × (WD^2^ - LD^2^).

### Electron microscopy

Mice were perfusion-fixed transcardially, and the thoracic aortas were isolated and fixed overnight at 4 ºC in 2.5% glutaraldehyde in cacodylate buffer with 5% sucrose. The samples were then embedded in epoxy resin. Ultrathin sections (80 nm) were prepared and double-stained with uranyl acetate and lead citrate. Autophagosomes were observed using a transmission electron microscope (H-7650, Hitachi, Japan).

### BrdU incorporation

MASMC proliferation was measured based on BrdU incorporation during DNA synthesis. After starvation for 24 h, cells in 96-well plates were treated with appropriate supplements before adding 10 mmol/L BrdU to the medium. Four hours later, the cells were fixed and incubated with an anti-BrdU antibody for 1 h at room temperature followed by incubation with a horseradish peroxidase-conjugated goat anti-IgG for 1 h. One hundred microliters of 3,3′,5,5′-tetramethylbenzidine (100 mmol/L) was then added as the substrate for horseradish peroxidase. BrdU incorporation was measured at 450 and 540 nm on a microplate reader (Bio-Tek, VT, USA).

### Co-immunoprecipitation

Immunoprecipitation was performed as we previously described 26,27. Cell lysates were immunoprecipitated with TMEM16A, p62, LC3B-I/II, bcl-2, Beclin-1 and VPS3 antibodies (those described for the western blotting protocol) using protein A/G agarose (sc-2003; Santa Cruz Biotechnology). The immunoprecipitates were washed with lysis buffer thrice and subjected to western blotting analysis. The bound proteins were immunoblotted with the indicated antibodies.

### VPS34 kinase assay

VPS34 kinase activity was assayed with a Mouse PI3KC3 Elisa Kit (Biomatik, Ontario, Canada). Assays were performed in aorta homogenates or cell lysates according to the manufacturer's instructions, and the product was detected spectrophotometrically at a wavelength of 450 nm on a microplate reader. Seven diluted standard concentrations (50, 25, 12.5, 6.25, 3.12, 1.56, and 0.78 ng/mL) were prepared, and 0 ng/mL was set as a blank. The concentration of VPS34 was calculated according to a standard curve and normalized to the total protein content.

### Statistical analysis

All data are expressed as the mean ± SEM and were analyzed using SPSS software, version 18.0 (SPSS Inc., IL, USA). n represents the number of independent experiments performed on different mice or different batches of cells. Comparisons between two groups were analyzed using a 2-tailed Student's *t*-test. One-way analysis of variance (ANOVA) followed by Bonferroni's multiple-comparison *post-hoc* test with a 95% confidence interval was employed when there were more than two groups. Correlation analyses were performed using the Pearson correlation test. *P* < 0.05 was considered to reflect a statistically significant difference.

## Results

### Autophagy levels positively correlated with AngII-induced vascular remodeling

**Figure 1:**
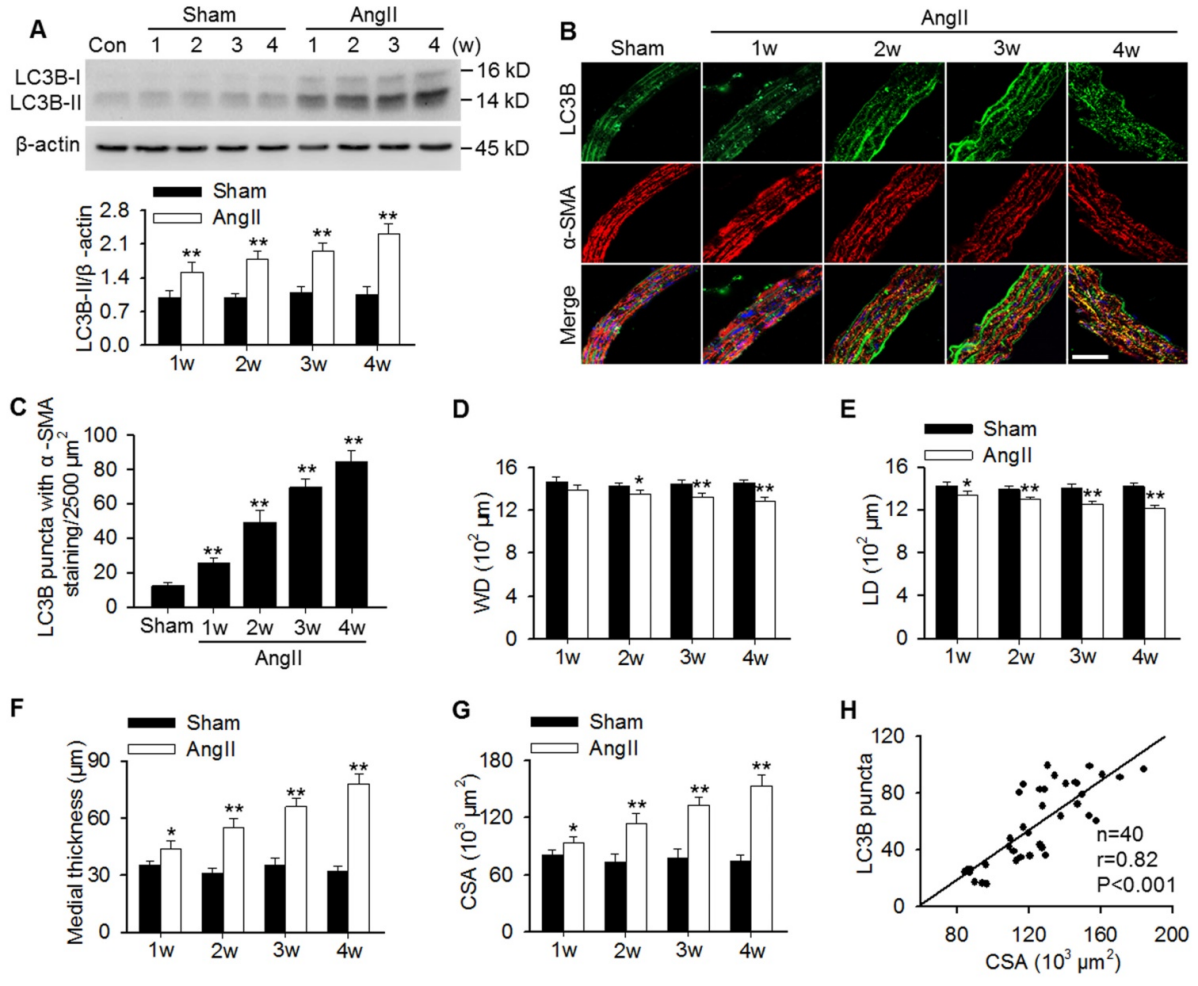
** Increased LC3B-positive puncta correlated positively with vascular remodeling during AngII-induced hypertension.** (A) Western blot analysis of LC3B-II expression in aortas from sham or AngII-infused mice at different time points after operation. **P < 0.01 vs. the corresponding sham group, Student's *t*-test. n = 10 mice/group. (B) Representative immunofluorescence staining of LC3B (green) and α-SMA (red) in thoracic aorta section. Nuclei were stained with DAPI. Scale bars, 20 µm. (C) Quantification of LC3B puncta. Five random fields (2,500 µm^2^/filed) were measured in one section. Statistical significance was determined by one-way ANOVA. **P < 0.01 vs. sham, n = 12 sections from six mice per group. (D-G) Structural parameters of mouse aorta from sham and hypertensive mice. WD, wall diameter; LD, lumen diameter; CSA, cross-sectional area. *P < 0.05, **P < 0.01 vs. the corresponding sham group, Student's *t*-test. n = 10 mice/group. (H) Correlation between changes in LC3B puncta and CSA values.

To examine whether autophagy is involved in AngII hypertension-induced vascular remodeling, protein expression of the autophagy marker LC3B was determined. The results showed that LC3B-II expression was gradually increased in aortas of AngII-induced hypertensive mice from 1 week post-operation (Figure 1A). In MASMCs, AngII also increased LC3B-II expression in a concentration-dependent manner (Figure S1A). Additionally, immunofluorescence analysis showed that AngII infusion showed a time-dependent increase in the number of LC3B-positive green puncta in the smooth muscle of aortas, as was identified by the smooth muscle marker α-SMA (Figure 1B and C). Although the decrease in wall and lumen diameter induced by AngII were mild, the difference was significant when compared to the corresponding sham groups (Figure 1D and E). Consequently, AngII infusion gradually increased the media thicknesses and the mean medial CSA value from 1 week post-operation (Figure 1F and G). Notably, correlation analysis demonstrated that the LC3B puncta positively correlated with the medial CSA values of thoracic aortas during AngII-induced hypertension (Figure 1H), indicating that autophagy may play a role in vascular remodeling.

### TMEM16A inhibited AngII-induced autophagy in aortic vessels

**Figure 2:**
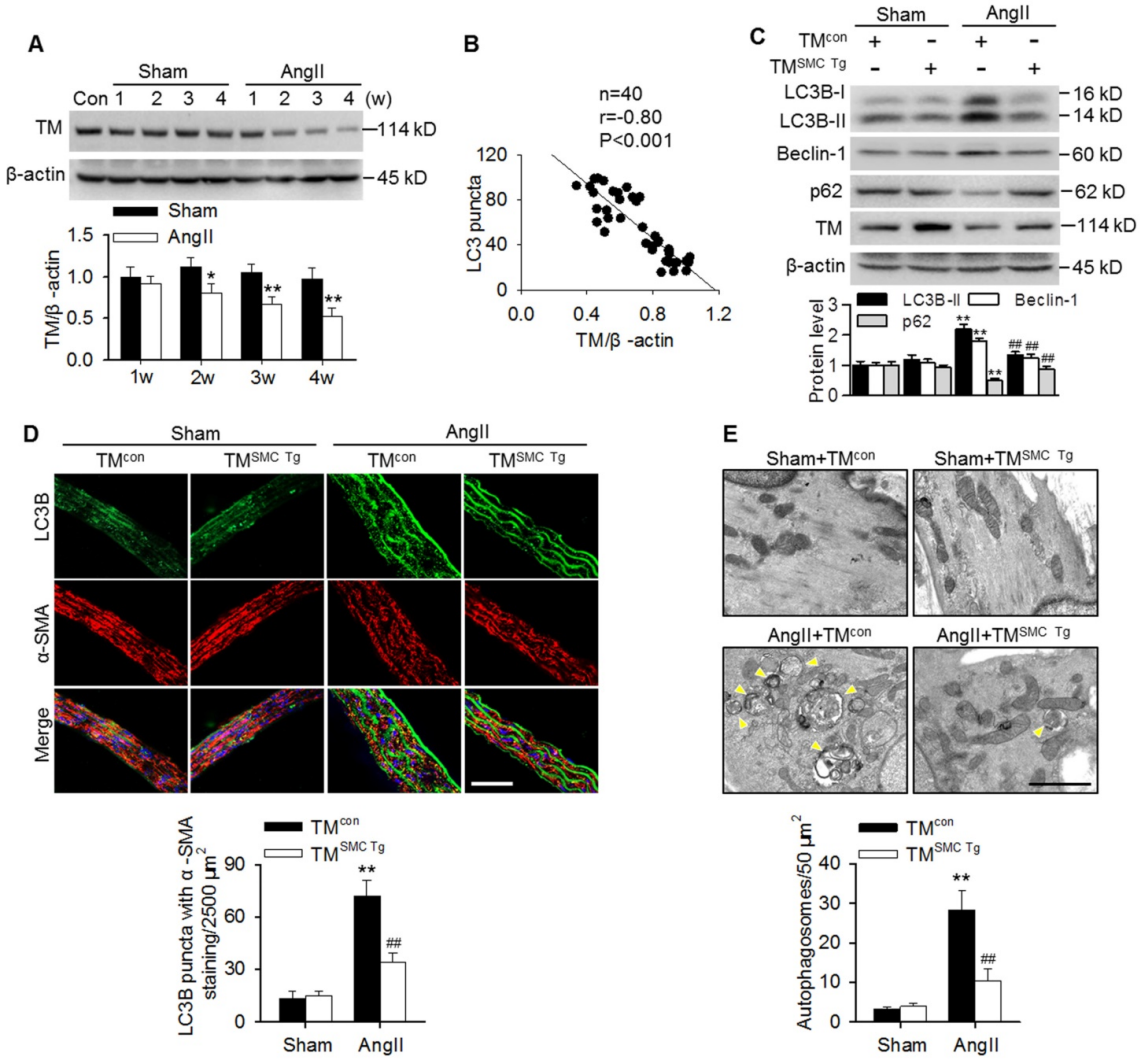
** TMEM16A inhibited AngII-induced autophagy in aortas.** (A) TMEM16A (TM) expression in aortas from AngII-induced hypertensive mice was lower than that in the corresponding sham mice from 2 weeks after operation. *P < 0.05, **P < 0.01 vs. corresponding sham group, Student's *t*-test. n = 10 mice/group. (B) Correlation between LC3B puncta and TMEM16A expression. (C) The expression levels of LC3B-II, Beclin-1 and p62 in aortas from TM^SMC Tg^ and TM^con^ mice after saline (sham) or AngII administration for 4 weeks. **P < 0.01 vs. sham + TM^con^; ##P < 0.01 vs. AngII + TM^con^, one-way ANOVA. n = 8 mice/group. (D) Representative immunofluorescence staining of LC3 (green) and α-SMA (red) in aortas. Scale bars, 20 µm. Quantification of LC3B puncta. Five random fields (2,500 µm^2^/filed) were measured in one section. **P < 0.01 vs. sham + TM^con^; ##P < 0.01 vs. AngII + TM^con^, one-way ANOVA. n = 14 sections from seven mice per group. (E) Transmission electron microscopy images showing autophagosomes (yellow arrowhead) in the medial layer of mouse aortas. Scale bars, 1 µm. Total autophagosome formation per 50 µm^2^ area in each section was quantitated. **P < 0.01 vs. sham + TM^con^; ##P < 0.01 vs. AngII + TM^con^, one-way ANOVA. n = 18 sections from six mice per group.

Similar to our previous results with basilar arteries 2,24, TMEM16A expression was gradually reduced in aortas of AngII hypertensive mice from 2 weeks post-operation compared with the corresponding sham mice (Figure 2A). This finding was consistent with our *in vitro* results showing that AngII also concentration-dependently inhibited TMEM16A expression in MASMCs (Figure S1B). Interestingly, the decreased TMEM16A expression negatively correlated with the numbers of LC3B-positive puncta in aortas from AngII hypertensive mice (Figure 2B). To explore the role of TMEM16A in hypertension-induced autophagy in aortic vessels, TMEM16A SMC-specific transgenic mice (TM^SMC Tg^) were generated and infused with AngII. Expression of the TMEM16A gene was detected by PCR genotyping (Figure S2A). Western blotting also confirmed that TMEM16A expression significantly increased in MASMCs of TM^SMC Tg^ mice, but not in MAECs (Figure S2B). Further, the AngII-induced upregulation of SBP was comparable between TM^con^ and TM^SMC Tg^ mice, suggesting that the SMC-specific TMEM16A transgene does not exert an effect on AngII-induced hypertension (Figure S2C). As shown in Figure 2C, LC3B-II, Beclin-1 and p62 expression in aortas were indistinguishable between sham TM^con^ and TM^SMC Tg^ mice. However, the AngII-induced increase in LC3B-II and Beclin-1 expression as well as the decrease in p62 expression were markedly inhibited in aortas from TM^SMC Tg^ mice compared with those from TM^con^ mice. Immunofluorescence staining revealed that SMC-specific TMEM16A overexpression markedly attenuated the AngII-induced increase in LC3B-positive puncta (Figure 2D). Further electron microscopic observations demonstrated an increased presence of autophagosomes in the medial layer of thoracic aortas from AngII-treated mice, compared with those from sham mice. TMEM16A upregulation significantly inhibited the accumulation of autophagosomes (Figure 2E). These data suggest an important role of TMEM16A expression in regulating vascular autophagy.

### TMEM16A blocked AngII-induced autophagic flux in MASMCs

**Figure 3:**
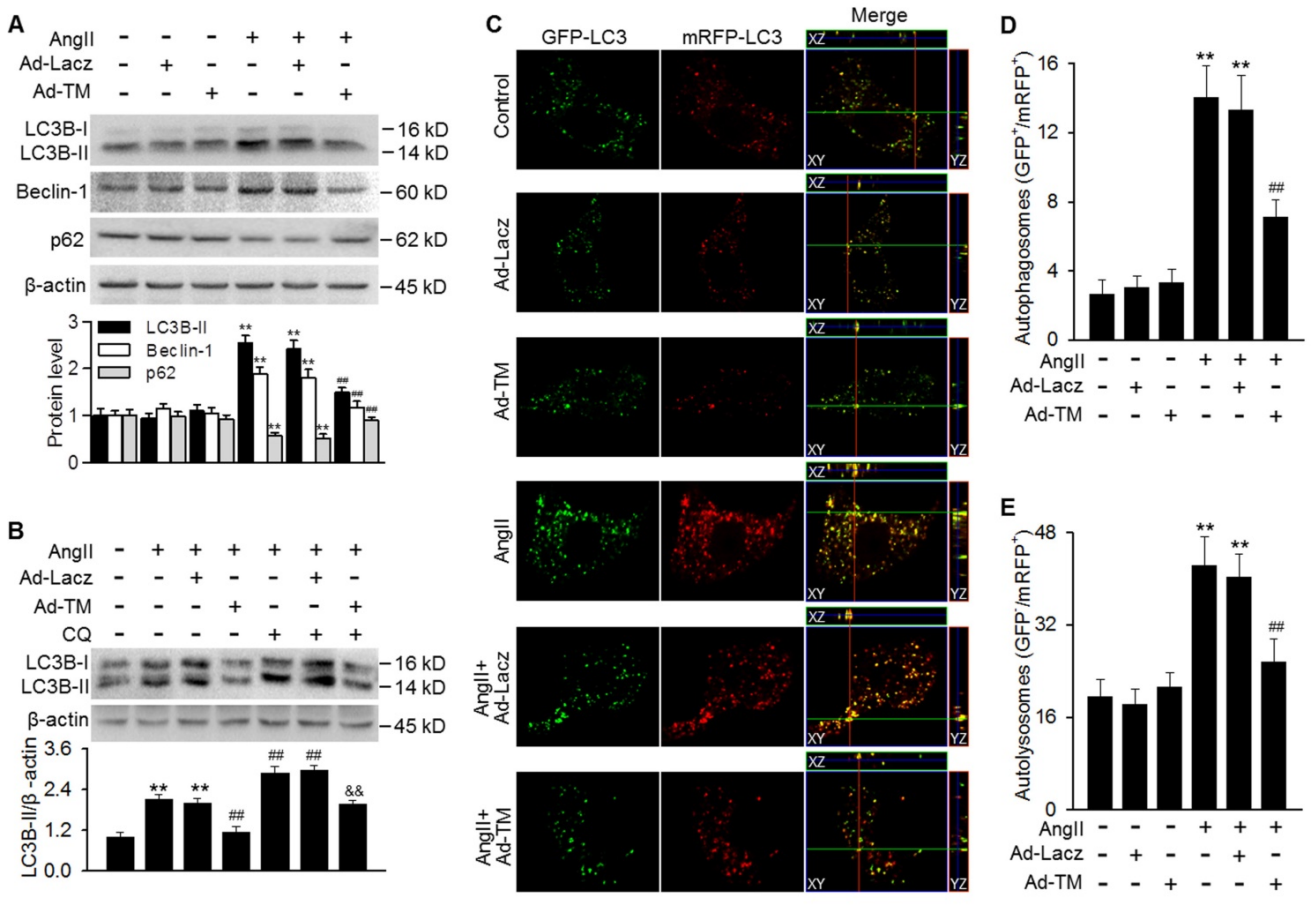
** TMEM16A overexpression blocked AngII-induced autophagic flux in MASMCs.** (A) Cells were infected with Lacz adenovirus (Ad-Lacz) or TMEM16A adenovirus (Ad-TM) at a multiplicity of infection (MOI) of 50 for 48 h followed by AngII administration (100 nmol/L) for 24 h. LC3B-II, Beclin-1 and p62 protein expression were measured. **P < 0.01 vs. control; ##P < 0.01 vs. AngII, one-way ANOVA. n = 6. (B) Ad-Lacz- or Ad-TM-infected MASMCs were incubated with chloroquine (CQ, 1 µmol/L) and AngII for 24 h. TMEM16A upregulation inhibited the accumulation of LC3B-II induced by chloroquine, following AngII stimulation. **P < 0.01 vs. control; ##P < 0.01 vs. AngII; &&P < 0.01 vs. AngII + CQ, one-way ANOVA. n = 6. (C) Cells were infected with tandem mRFP-GFP-LC3 adenovirus and Ad-TM or Ad-Lacz for 48 h prior to AngII treatment for 24 h. Representative cells were examined for fluorescence by confocal microscopy. Scale bars, 10 µm. Cells were stained with DAPI to label cell nuclei. (D and E) Quantification of autophagosomes (GFP^+^/RFP^+^) (D) and autolysosome numbers (GFP^-^/RFP^+^) (E). The average number of puncta per cell was calculated from 100 cells in each experiment (n = 4 independent experiments). **P < 0.01 vs. control; ##P < 0.01 vs. AngII. Statistical significance was determined by one-way ANOVA.

Our findings showing the inhibition of AngII-induced autophagy in TM^SMC Tg^ mice promoted us to examine whether TMEM16A plays a similar role *in vitro*. Infecting MASMCs with TMEM16A adenovirus markedly increased TMEM16A expression, compared with control group (Figure S3A). TMEM16A overexpression significantly inhibited the AngII-induced increase in LC3B-II and Beclin-1 expression as well as the decrease in p62 expression (Figure 3A). To distinguish whether the increased LC3B-II and Beclin-1 levels were due to increased autophagosome formation or impaired autophagosome-lysosome fusion, we examined the effect of chloroquine (a lysosome inhibitor) on LC3B-II expression. The results showed further enhancement of LC3B-II expression in AngII-treated MASMCs following inhibition of lysosomal activity. However, the effect of chloroquine on LC3B-II expression was markedly reversed by TMEM16A overexpression, suggesting that TMEM16A inhibited autophagosome formation (Figure 3B). To confirm the effects of TMEM16A on autophagic flux, a tandem mRFP-GFP-LC3 adenovirus was employed for fluorescence analysis. GFP fluorescence is optimal under a higher pH value and can be quenched more quickly in a lysosomal acid environment, compared with mRFP, which enables us to monitor autophagosomes and autolysosomes by detecting yellow puncta (GFP^+^/mRFP^+^) and red puncta (GFP^-^/mRFP^+^), respectively 28. MASMCs treated with AngII showed abundant yellow and red puncta. However, ectopic overexpression of TMEM16A significantly decreased the number of both yellow and red puncta compared to AngII treatment alone (Figure 3C-E), demonstrating that TMEM16A attenuates AngII-induced autophagy via inhibiting autophagic flux.

### Downregulation of TMEM16A promoted AngII-induced autophagic flux

**Figure 4:**
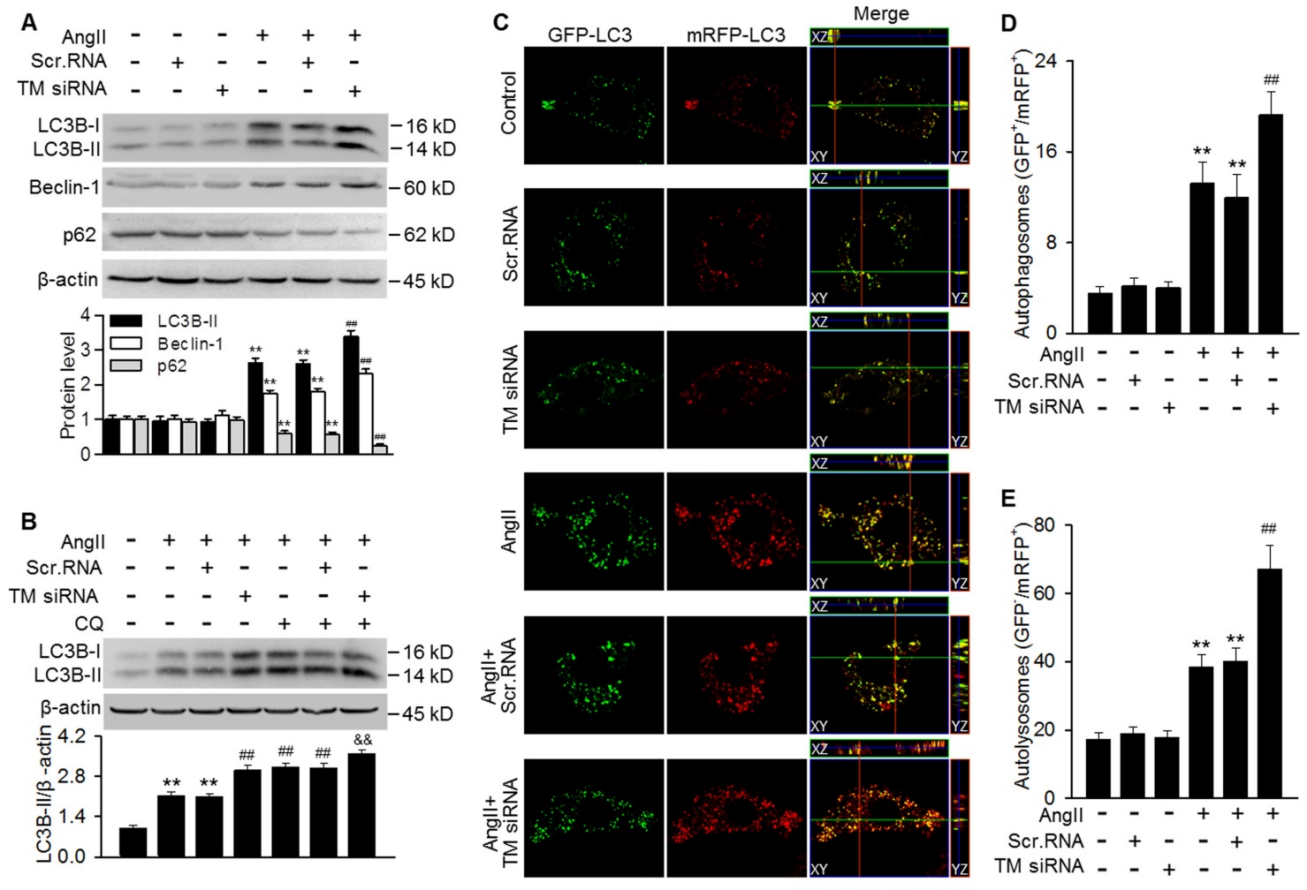
** Autophagosome accumulation induced by AngII was potentiated by TMEM16A downregulation.** (A) Cells were treated with TMEM16A siRNA (TM siRNA, 25 nmol/L) or scrambled siRNA (scr.RNA) for 48 h before AngII stimulation. The protein expression of LC3B-II, Beclin-1 and p62 were measured. **P < 0.01 vs. control; ##P < 0.01 vs. AngII, one-way ANOVA. n = 6. (B) MASMCs were transfected with TM siRNA and then treated with CQ and AngII for 24 h, after which LC3B-II expression was measured. **P < 0.01 vs. control; ##P < 0.01 vs. AngII; &&P < 0.01 vs. AngII + CQ, one-way ANOVA. n = 6. (C) mRFP-GFP-LC3 expressing cells were treated with scr.RNA or TM siRNA, with or without AngII stimulation. Images were acquired with a confocal microscope. Scale bars, 10 µm. (D and E) Autophagosome (GFP^+^/RFP^+^) (D) and autolysosome (GFP^-^/RFP^+^) (E) numbers were calculated, using 100 cells from each experiment and averaged. **P < 0.01 vs. control; ##P < 0.01 vs. AngII, one-way ANOVA. n = 4.

To further clarify the role of TMEM16A in regulating autophagy, TMEM16A expression was knocked down with siRNA in MASMCs. Successful downregulation of TMEM16A was confirmed by western blotting using an anti-TMEM16A antibody (Figure S3B). The results showed that TMEM16A knockdown further potentiated the AngII-induced increase in LC3B-II and Beclin-1 expression and decrease in p62 expression (Figure 4A). Moreover, the effect of chloroquine on LC3B-II expression was augmented in TMEM16A siRNA-treated cells (Figure 4B), indicating that TMEM16A downregulation further promoted autophagosome maturation. Similar results were observed in terms of autophagic flux with the tandem mRFP-GFP-LC3 adenovirus. Compared with AngII treatment alone, TMEM16A knockdown markedly increased the numbers of both yellow and red puncta (Figure 4C-E), suggesting that TMEM16A knockdown potentiates AngII-induced autophagy.

### TMEM16A inhibited AngII-induced VPS34 activity

**Figure 5:**
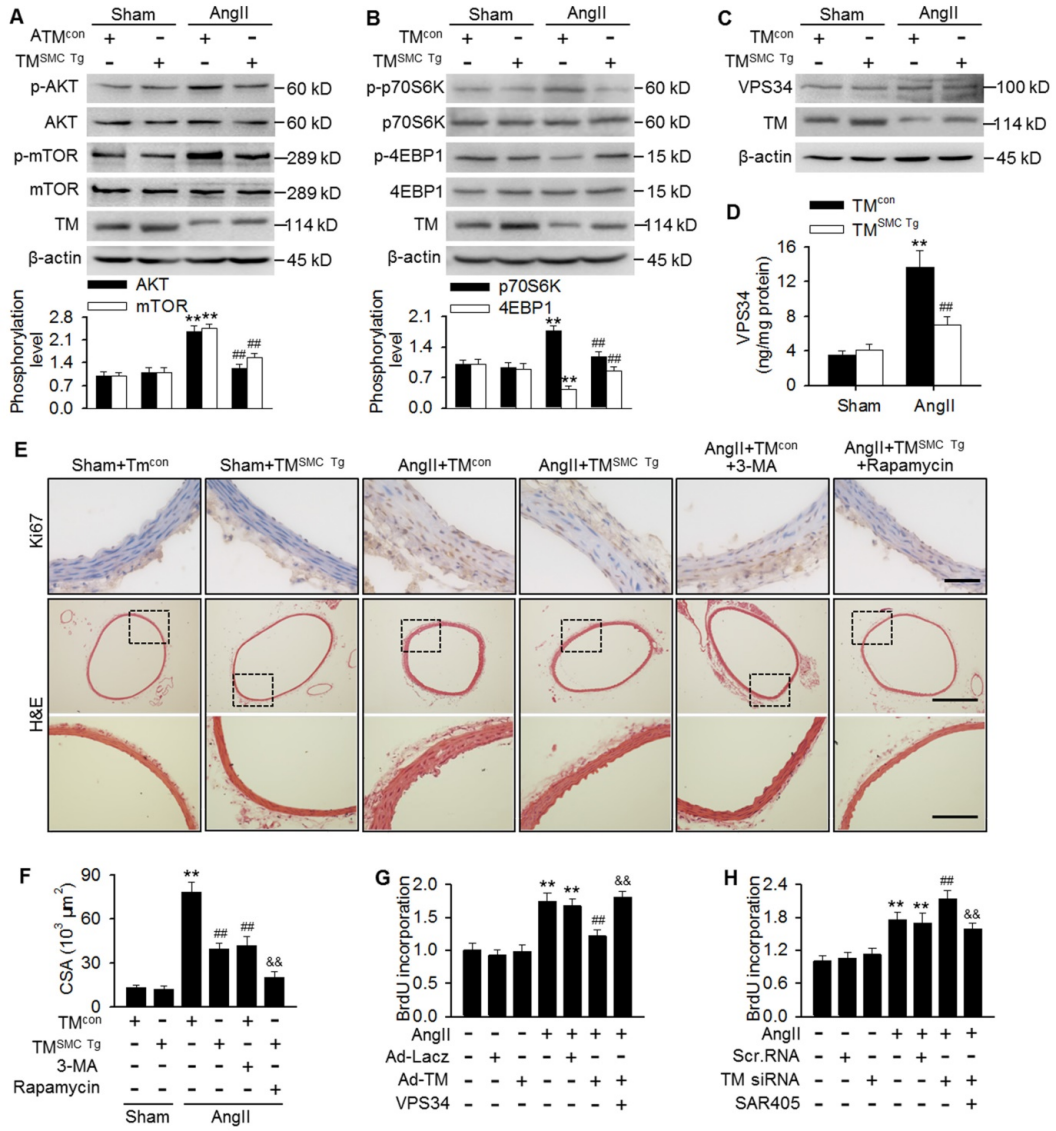
** TMEM16A ameliorated autophagy-meditated vascular remodeling by reducing VPS34 activity.** (A-C) Western blot analysis of the phosphorylation of AKT, mTOR (A), p70S6K, and 4EBP1 (B) and the expression of VPS34 (C) in aortas from TM^SMC Tg^ and TM^con^ mice following AngII infusion for 4 weeks. (D) Bar charts showing VPS34 kinase activity in aorta homogenates. **P < 0.01 vs. sham + TM^con^; ##P < 0.01 vs. AngII + TM^con^. Statistical significance was determined by one-way ANOVA. n = 6 mice/ group. (E) TM^con^ or TM^SMC Tg^ mice were injected intraperitoneally with 3-MA (100 µg/kg) or rapamycin (1 mg/kg), respectively, at 2 weeks after AngII infusion, and then continuously dosed once per day for 2 weeks. Representative images of Ki67 staining and hematoxylin-eosin staining of aortic sections are shown. Scale bars, 25 µm (upper panel), 200 µm (middle panel) or 50 µm (lower panel). (F) Quantification of cross-sectional areas (CSAs). **P < 0.01 vs. sham + TM^con^; ##P < 0.01 vs. AngII + TM^con^; &&P < 0.01 vs. AngII + TM^SMC Tg^, one-way ANOVA. n = 6 mice/ group. (G) Cells were infected with Ad-Lacz or Ad-TM for 48 h and then transfected with VPS34 plasmid in the presence of AngII for 24 h. Cell proliferation was determined by performing BrdU assays. (H) Proliferation of MASMCs transfected with scr.RNA or TM siRNA following AngII and SAR405 (1 µmol/L) treatment for 24 h. **P < 0.01 vs. control; ##P < 0.01 vs. AngII; &&P < 0.01 vs. AngII + Ad-TM or AngII + TM siRNA, one-way ANOVA. n = 6.

mTOR pathway inhibition has been reported to play an important role in autophagy initiation 12,25,29. To determine the mechanisms by which TMEM16A regulates autophagy, we investigated the effect of TMEM16A on the mTOR pathway. Unexpectedly, phosphorylation of AKT, mTOR, and the downstream target p70S6K were increased significantly, while 4-EBP1 phosphorylation was inhibited in aortas from AngII-treated mice, as compared with sham mice. In contrast, SMC-specific TMEM16A overexpression markedly inhibited mTOR pathway activation (Figure 5A and B). This finding was further confirmed by *in vitro*. TMEM16A overexpression attenuated, whereas TMEM16A siRNA augmented AngII-induced activation of the mTOR pathway (Figure S4A-D). These results indicate that the autophagy mediated by TMEM16A may be independent of the mTOR machinery. In addition to the mTOR pathway, VPS34 is also a well-characterized regulator of autophagy 28. VPS34 expression was similar in aortas from AngII-induced hypertensive TM^SMC Tg^ and TM^con^ mice (Figure 5C). Similarly, neither TMEM16A overexpression nor TMEM16A inhibition altered VPS34 expression in the presence or absence of AngII treatment (Figure S5A and B). Nevertheless, AngII infusion markedly increased the kinase activity of VPS34, which was inhibited by SMC-specific TMEM16A overexpression (Figure 5D). Consistently, in MASMCs, TMEM16A upregulation attenuated the AngII-induced increase in VPS34 activity, and the opposite result was observed after TMEM16A knockdown (Figure S5C and D).

Considering the involvement of TMEM16A and autophagy in vascular remodeling and the capacity of TMEM16A to regulate autophagy, we thus explored the possibility that autophagy may be essential for TMEM16A-mediated vascular SMC proliferation and remodeling. Results showed that in thoracic aortas, the number of Ki67-postive cells and medial CSA values were similar in TM^con^ and TM^SMC Tg^ mice, under basal conditions. However, AngII infusion dramatically increased the level of Ki67 staining as well as the medial CSA value, which was inhibited in TM^SMC Tg^ mice. Further, administration of 3-MA, an autophagy inhibitor that targets VPS34, markedly inhibited AngII-induced VSMC proliferation and vascular remodeling, similar to what was observed in AngII-treated TM^SMC Tg^ mice (Figure 5E and F). The *in vitro* results further demonstrated that inhibition of autophagy served to attenuate AngII-induced VSMC proliferation (Figure S6); thereby confirming that autophagy was indeed involved in VSMC proliferation and vascular remodeling. Of note, administration of rapamycin, an autophagy stimulator through inhibiting mTOR signaling, did not reverse the inhibitory effect of TMEM16A upregulation on VSMC proliferation and vascular remodeling, but rather augmented this effect (Figure 5E and F). Additionally, AngII-induced MASMC proliferation was markedly attenuated by TMEM16A overexpression and potentiated by TMEM16A knockdown (Figure 5G and H). Overexpression of VPS34 ablated the inhibitory effect of TMEM16A upregulation on cell proliferation induced by AngII (Figure 5G). Furthermore, pharmacological inhibition of VPS34 with a more highly selective inhibitor (SAR405) largely abrogated the increase in cell proliferation caused by TMEM16A downregulation (Figure 5H). Collectively, these results indicate that inhibition of VPS34-mediated autophagy underlies the effects of TMEM16A on vascular SMC proliferation and remodeling.

### TMEM16A sequestered p62 from p62/Bcl-2/Beclin-1/VPS43 complexes and inhibited autophagy initiation

**Figure 6:**
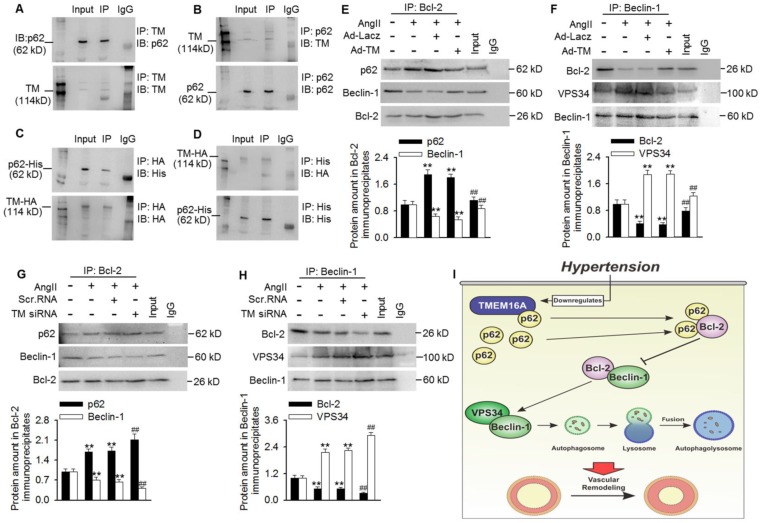
** TMEM16A bound with p62 and modulated p62-Bcl-2-Beclin-1-VPS43 complex formation.** (A and B) Immunoprecipitation (IP) followed by immunoblotting (IB) in MASMC lysates showing presence of p62 in TMEM16A immunoprecipitates (A), and reciprocal immunoprecipitation showing the presence of TMEM16A in p62 immunoprecipitates (B). (C and D) MASMCs were co-transfected with vectors expressing TMEM16A tagged with HA and RFP, and p62 tagged with His. Western blot analysis for His (C) or HA (D) after immunoprecipitation with an HA or His antibody, respectively. n=5. (E-H) The cells were infected with Ad-Lacz or Ad-TM adenovirus (E and F, respectively), or transfected with scr.RNA or TM siRNA (G and H, respectively) for 48 h and then treated with AngII for another 24 h. Lysates were immunoprecipitated with a Bcl-2 (E and G) or Beclin-1 (F and H) antibody and immunoblotted with the indicated antibodies. **P < 0.01 vs. control; ##P < 0.01 vs. AngII, one-way ANOVA. n = 7. (I) Schematic representation of the study findings. TMEM16A bound with p62 and sequestered it from the p62-Bcl-2 complex. AngII-induced TMEM16A downregulation and led to reduced TMEM16A-p62 association and facilitated the binding of p62-Bcl-2, which limited formation of Bcl-2-Beclin-1 complexes. Finally, the enhanced Beclin-1-VPS34 association increased the kinase activity of VPS34 and initiated autophagy, leading to VSMC hyperplasia and remodeling.

To investigate how TMEM16A attenuated VPS34 activity and subsequent autophagic flux, we investigated the role of TMEM16A in regulating autophagy initiation. After a series of co-immunoprecipitation tests, we did not detect any interactions between TMEM16A and Beclin-1, LC3B, Bcl-2, or VPS34 in MASMCs (Figure S7). Surprisingly, TMEM16A co-immunoprecipitated with p62, and a reciprocal interaction was also observed by immunoprecipitating with an anti-p62 antibody in MASMCs and aortas lysates (Figure 6A and B and Figure S8A). Consistently, TMEM16A interacted with exogenous p62 in cells transfected with HA-RFP-TMEM16A and His-p62 (Figure 6C and D). Additionally, subcellular localization, as observed via confocal microscopy in MASMCs showed that TMEM16A and p62 were colocalized in MASMCs (Figure S8B). It was previously reported that p62 bound Bcl-2 and prevented formation of Bcl-2/Beclin-1 complexes 24,25. Disruption of the Bcl-2-Beclin-1 interaction promoted the Beclin-1-VPS34 association and increased Beclin-1-related VPS34 activity 29. Based on these observations, we next examined associations among these related proteins. Treating MASMCs with AngII increased the binding of p62-Bcl-2 and VPS34-Beclin-1, respectively, but reduced the amount of Bcl-2 that co-immunoprecipitated with Beclin-1 (Figure 6E-H). TMEM16A overexpression significantly limited the increased formation of p62-Bcl-2 and VPS34-Beclin-1, but reversed the decrease in Bcl-2-Beclin-1 binding (Figure 6E and F). In contrast, TMEM16A suppression enhanced the binding of p62-Bcl-2 and VPS34-Beclin-1, and further disrupted the formation of Bcl-2-Beclin-1 complex (Figure 6G and H). These protein interactions were further confirmed by immunofluorescence colocalization (Figure S8C). This suggests that TMEM16A regulates VPS34 activity at least partially via the four-way interaction between p62, Bcl-2, Beclin-1, and VPS34.

## Discussion

Here, we explored links between TMEM16A expression and autophagy-mediated VSMC proliferation during vascular remodeling. Our data identified TMEM16A as a critical molecule in AngII-induced autophagy and, thus, vascular tone regulation. During AngII-induced hypertension, TMEM16A expression was decreased in aortic vessels, which attenuated TMEM16A-p62 binding. In turn, disrupting TMEM16A-p62 complex formation increased the binding of p62-Bcl-2 and subsequently blunted Bcl-2-Beclin-1 complex formation, leading to enhanced Beclin-1-VPS34 interaction and Beclin-1-associated VPS34 activity. Such cooperation induced autophagy in VSMCs. As such, whereas TMEM16A insufficiency was associated with autophagy-mediated VSMCs proliferation, it may simultaneously protect against vascular remodeling during hypertension (Figure 6I).

In the cardiovascular system, autophagy plays dual roles through adaptive or maladaptive regulation 4,9,14,15. Autophagy activation was associated with hypoxia-induced pulmonary arterial SMC (PASMC) proliferation, and 3-MA inhibited the autophagy-mediated cell proliferation 29. Similarly, another autophagy inhibitor (chloroquine) also prevented the progression of experimental pulmonary hypertension 10. Furthermore, direct disruption of the autophagy pathway using Atg-5 siRNA limited rat PASMC proliferation 32. Conversely, LC3B lacking potentiated hypoxia-induced PAMSC proliferation 33. A recent work revealed that autophagy deficiency in VSMCs contributed atherosclerosis and restenosis 13. This outcome was also attributed to macrophage apoptosis and, thus, the atherosclerotic plaques rupture 11. Despite these inconsistent conclusions regarding the roles of autophagy in cardiovascular diseases, much of the data collected were dependent upon the experimental conditions or cell types. Autophagy may serve a protective role under physiological conditions to maintain normal cardiovascular function, but it also contributes disease development under pathological conditions when unstrained 4. Although AngII may induce autophagy in rat VSMCs 7, no report has described the roles of autophagy in the pathogenic effects of AngII on the vascular system, such as vascular remodeling.

Our results showed that AngII induced LC3B-II and Beclin-1 expression in both aortas and MASMCs, which was consistent with a previous study 7. It is noteworthy that enhanced expression of LC3B-II and Beclin-1 cannot fully indicate the presence of increased autophagic activity; as such expression differences may be consequences of reduced autophagosome degradation, which would lead to either LC3B-II accumulation or Beclin-1 transcription through a feedback loop 28. However, this did not occur in AngII-stimulated VSMCs, since inhibition of autophagosome degradation using chloroquine further aggravated AngII-induced LC3B-II accumulation, confirming the increased autophagic activity in VSMCs after AngII challenge. AngII induced autophagosome and autolysosome accumulation with a tandem mRFP-GFP-LC3 adenovirus, further supporting the possibility that autophagic flux increased. More importantly, the increase of LC3B puncta positively correlated with the medial CSA values of aortas in AngII-induced hypertensive mice, indicating that increased autophagy may have been involved in vascular remodeling during hypertension. Indeed, the autophagy inhibitor 3-MA significantly ameliorated AngII hypertension-induced vascular remodeling. Collectively, these data suggest that AngII can induce autophagosome formation and autophagic flux in VSMCs, and subsequently promote cell proliferation and vascular remodeling.

Although activation of CaCC has been reported to enhance the smooth muscle contractile response of blood vessels 32, the unchanged blood pressure in TM^SMC Tg^ mice seems to abnegate the results of a vascular-specific TMEM16A ablation mouse study, in which lower blood pressure in response to AngII was observed 35. Moreover, an *ex vivo* study further indicated that knockdown of TMEM16A in cultured cerebral arteries inhibited pressure-induced vasoconstriction 23. Since TMEM16A-indued enhancement of CaCC activity can be counterbalanced by other factors, the effect of TMEM16A overexpression may be inconsistent with TMEM16A knockout. Moreover, according to the report by Heinze et al. 35, we designed our targeting vector for vascular-specific TMEM16A knockout mice by flanking TMEM16A exon 12. The truncated TMEM16A protein produced by this animal model could be recognized by a novel TMEM16A antibody against the N-terminal region. However, this mutant did not elicit CaCC, the thus, the potential role of TMEM16A in regulating blood pressure remains unclear. Additionally, in the previous study 35, no structural changes were observed in blood vessels in vascular-specific TMEM16A knockout mice, while our study showed that TMEM16A overexpression markedly ameliorated vascular remodeling. This may suggest distinct difference in the effect between TMEM16A overexpression and knockdown on vascular remodeling as well as on blood pressure.

Consistent with our previous studies demonstrating decreased TMEM16A expression in basilar arteries from hypertensive animals 2,24; we observed lower TMEM16A expression in thoracic aortas from AngII-induced hypertensive mice. Our *in vitro* results further suggested that AngII inhibited TMEM16A expression in MASMCs, in full agreement with previous findings with human aortic SMCs and basilar artery SMCs (BASMCs) 2,3,21, 24. Previously, we demonstrated that TMEM16A negatively regulated BASMC proliferation and basilar artery remodeling by inhibiting cell cycle progression 2. Zhang et al. showed that TMEM16A inhibited VSMC proliferation by forming a positive-feedback loop with myocardin 3. Consistently, we found that the remodeling of thoracic aortas by AngII was markedly decreased in TMEM16A SMC-specific transgenic mice. TMEM16A overexpression inhibited the AngII-induced decrease in MASMC proliferation, whereas TMEM16A knockdown led to the opposite results. Nonetheless, apart from the above observations 2,3, the mechanisms whereby TMEM16A prevents vascular remodeling are still poorly understood. Based on the correlation between autophagy and vascular remodeling, we hypothesized that the effect of TMEM16A on the vascular tone may be associated with autophagy regulation. The extent of reduced TMEM16A expression in aortas correlated negatively with the number of LC3B puncta. We further demonstrated that SMC-specific TMEM16A overexpression significantly inhibited the AngII-induced increase in autophagy, whereas TMEM16A downregulation in MASMCs potentiated the effect of AngII. To our knowledge, this report is the first to demonstrate that TMEM16A can inhibit AngII-induced autophagy in VSMCs. Our current findings, together with those from previous studies, indicate the particular importance of chloride channels in autophagy regulation 17-20.

mTOR is one of the major signaling pathways that regulate autophagy 12,29. PI3K/AKT-signaling activation (upstream of mTOR) contributes autophagy inhibition and cell proliferation 25,29. Unexpectedly, in our study, the AKT/mTOR pathway was not involved in AngII-induced autophagy, as AngII induced rather than inhibited AKT/mTOR pathway activation. This activation was much more pronounced in TMEM16A siRNA-treated cells, but was markedly alleviated after TMEM16A overexpression. Moreover, mTOR inhibition by rapamycin augmented instead of reversed the effect of TMEM16A upregulation on VSMC proliferation and vascular remodeling. These results are consistent with the clinical use of rapamycin and its derivatives (e.g., sirolimus and everolimus) for treating restenosis and atherosclerosis via the inhibition of VSMC proliferation by reducing the expression of key cell cycle-related proteins 12,36. Indeed, we previously reported that TMEM16A prevented AngII-induced BASMC proliferation by inhibiting cell cycle progression 2. Thus, our data suggest that the effect of mTOR inhibition on AngII-induced vascular remodeling may be independent of autophagy regulation. Interestingly, the autophagy inhibitor 3-MA, which targets VPS34 25, attenuated AngII-induced vascular remodeling in our study, and we thereby investigated the role of VPS34. TMEM16A overexpression significantly inhibited the AngII-induced increase in VPS34 activity, although VPS34 protein expression was similar among the experimental groups. The inhibitory effect of TMEM16A upregulation on VSMC proliferation was abolished by VPS34 restoration, while the enhanced proliferation induced by TMEM16A downregulation was completely reversed by the VPS34 inhibitor. Collectively, these findings demonstrate that the reduced VPS34 activity contributed the inhibitory effect of TMEM16A on autophagy-mediated vascular SMC proliferation and remodeling.

VPS34, also known as class-III PI3K (PI3KC3), is a member of PI3K family 30. This kinase participates in autophagy initiation, whereas the AKT/mTOR pathway is primarily activated by class-I PI3K 25,31. The Beclin-1-VPS34 complex plays a crucial role in autophagosome nucleation 28. The kinase activity of this complex is negatively regulated by Bcl-2, which binds with Beclin-1 and disturbs the formation of Beclin-1-VPS34, thereby inhibiting autophagosome formation 31. To better understand how TMEM16A regulates VPS34 activity, it may be necessary to identify TMEM16A binding partners. Consistent with our previous study in BASMCs 37, we found that TMEM16A interacted with p62 in MASMCs. Additionally, previous reports showed that p62 can bind with Bcl-2 and subsequently decrease the binding of Bcl-2-Beclin-1 26,27. Based on these reports, we speculated that TMEM16A influences interactions between p62, Bcl-2, Beclin-1, and VPS34, and coordinately regulates autophagy in VSMCs. TMEM16A overexpression attenuated the AngII-induced increase in p62-Bcl-2 association and led to Bcl-2 release and Bcl-2-Beclin-1 formation, thereby sequestering Beclin-1 from the Beclin-1-VPS34 complex. However, TMEM16A knockdown ultimately promoted Beclin-1-VPS34 complex formation. The present data indicate that the amount of TMEM16A is critical for autophagy initiation, which may explain why TMEM16A downregulation in AngII-induced hypertension increased VPS34 activity and triggered autophagy-mediated vascular remodeling.

In conclusion, TMEM16A ameliorated AngII-induced vascular remodeling by inhibiting autophagy-mediated VSMC proliferation via modulation of a four-way interaction between p62, Bcl-2, Beclin-1, and VPS34, which consequently decreased VPS34 activity. These findings provide novel insights into the mechanism whereby TMEM16A attenuates vascular remodeling and suggest a promising targeted therapy for this disease.

## Supplementary Material

Supplementary figures.

## Abbreviations

TMEM16A: Transmembrane member 16A
SMC: smooth muscle cell
MASMC: mouse aortic smooth muscle cell
AngII: angiotensin II
VPS34: vacuolar protein sorting 34
DMEM: Dulbecco's modified Eagle's medium
FBS: fetal bovine serum
ECGF: endothelial cell growth factor
BrdU: 5-bromo-2'-deoxyuridine
BASMC: basilar artery smooth muscle cell
TM^SMC Tg^: smooth muscle cell-specific transgenic mice
MAECs: mouse aortic endothelial cells
mRFP: monomeric red fluorescent protein
GFP: green fluorescent protein
WD: wall diameter
LD: lumen diameter
CSA: cross-sectional area
