# Supplementary material for: TMEM16A ameliorates vascular remodeling by suppressing autophagy via inhibiting Bcl-2-p62 complex formation
Source: Theranostics. 2020 Mar 4;10(9):3980–93. doi: 10.7150/thno.41028 (PMC7086348; doi:10.7150/thno.41028)
Supplement: Supplementary file 1 — Supplementary figures. [file thnov10p3980s1.pdf]

Supplementary figures

Figure S1

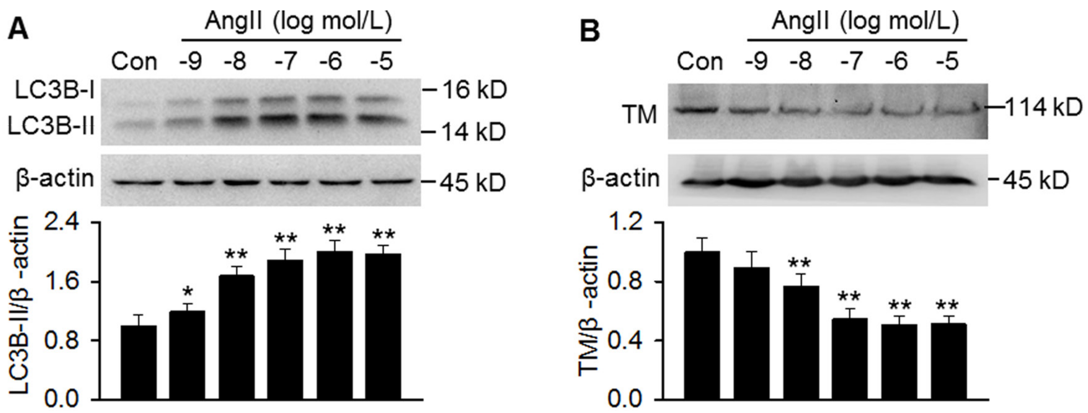

Figure S1. Effects of Angiotensin II (AngII) on LC3B-II and TMEM16A expression in MASMCs. (A and B) Cells were treated with various concentrations of AngII (10^{-9}, 10^{-8}, 10^{-7}, 10^{-6}, and 10^{-5} mol/L) for 24 h. The expression of LC3B-II (A) and TMEM16A (TM) (B) were determined by western blotting. *P < 0.05, **P < 0.01 vs. control, Student's *t*-test. n = 6.

Figure S2

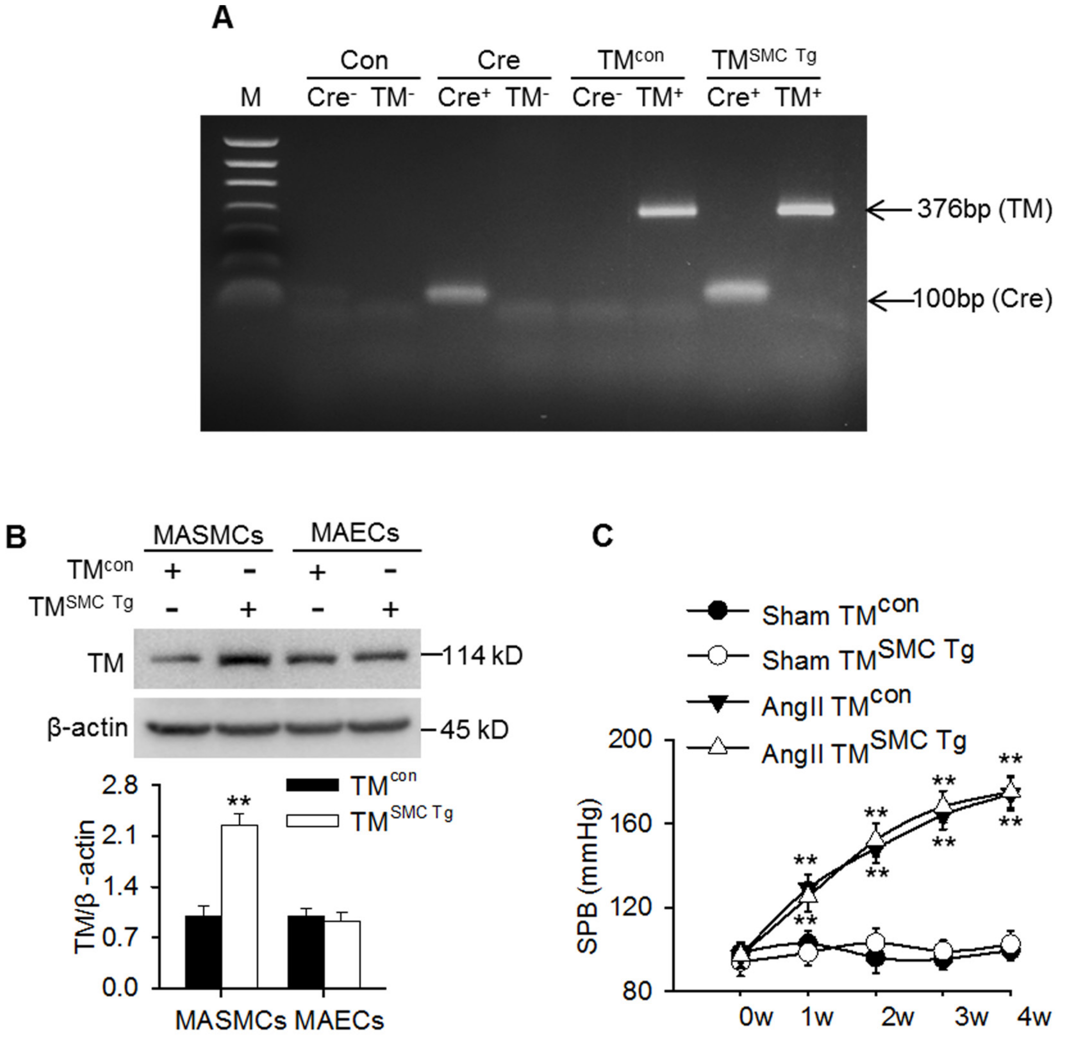

Figure S2. Identification of TMEM16A smooth muscle cell (SMC)-specific transgenic mice (TM$^{SMC\ Tg}$). (A) Mice were identified by PCR-based amplification of genomic DNA using primers specific for transgene-TMEM16A and SM22α-Cre, respectively. Mice containing transgene-TMEM16A and SM22α-Cre were defined as TM$^{SMC\ Tg}$ mice. Mice containing transgene-TMEM16A, but not SM22α-Cre were defined as TM^{con} mice. By design, the wild-type allele was not amplified, and the TM^{con} mice produced a band at 376 base pairs (bp). Cre^{+} mice showed a 100-bp band, whereas no band was observed with Cre^{-} mice. (B) Western blot analysis of TMEM16A expression in mouse aortic SMCs (MASMCs) and mouse aortic endothelial cells (MAECs) from TM$^{SMC\ Tg}$ and TM^{con} mice. $^{**}P < 0.01$ vs. TM^{con}, Student's *t*-test. n = 6 mice/group. (C) AngII infusion increased the systolic blood pressure (SBP) to the same level in both TM^{con} mice and TM$^{SMC\ Tg}$ mice. SBP was

measured before and after chronic Ang II infusion for 4 wk. **P < 0.01 vs. TM^{con}, one-way ANOVA. n = 8 mice/group.

Figure S3

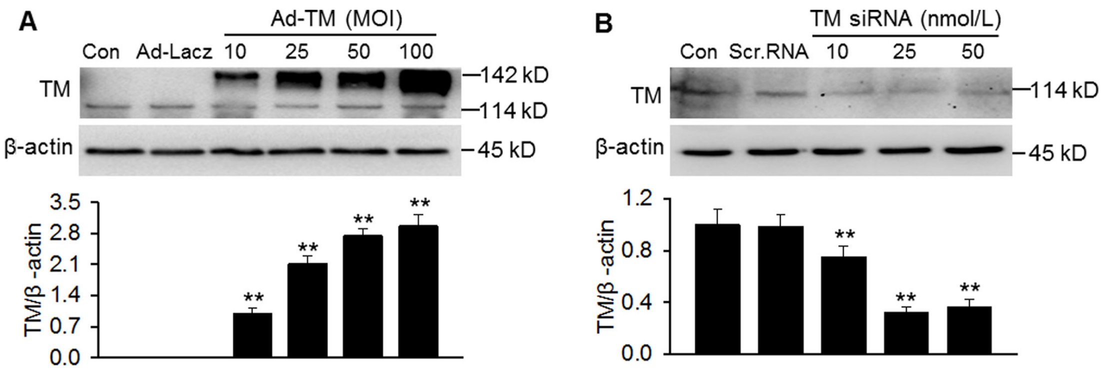

Figure S3. Effect of TMEM16A adenovirus or siRNA on TMEM16A expression. (A) The cells were infected with different MOI of TMEM16A adenovirus (Ad-TM) for 48 h. TMEM16A expression was measured. (B) Western blotting showed that TMEM16A siRNA (TM siRNA) transfection for 48 h decreased TMEM16A (TM) protein expression in MASMCs. **P < 0.01 vs. TM^{com}, Student's *t*-test. n = 4.

Figure S4

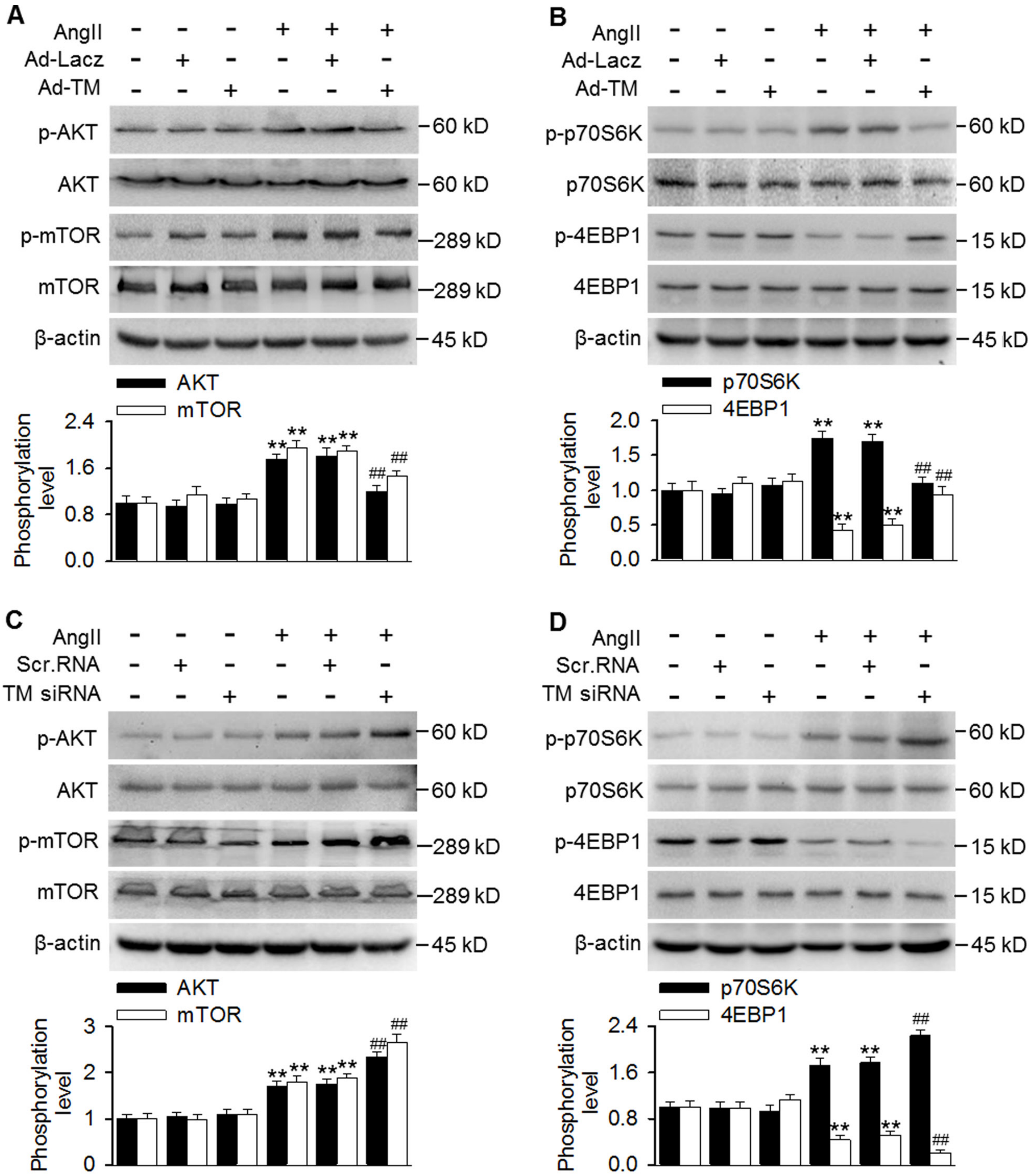

Figure S4. TMEM16A overexpression inhibited AngII-induced AKT/mTOR pathway activation in MASMCs. (A–D) MASMCs were infected with Ad-Lacz or Ad-TM (A and B, respectively), or transfected with scr.RNA or TM siRNA (C and D, respectively) for 48 h followed by AngII administration (100 nmol/L) for 24 h. The phosphorylation of AKT, mTOR (A and C), p70S6K, and 4EBP1 (B and D) were determined by western blotting. **P < 0.01 vs. control; ##P < 0.01 vs. AngII, one-way ANOVA. n = 5.

Figure S5

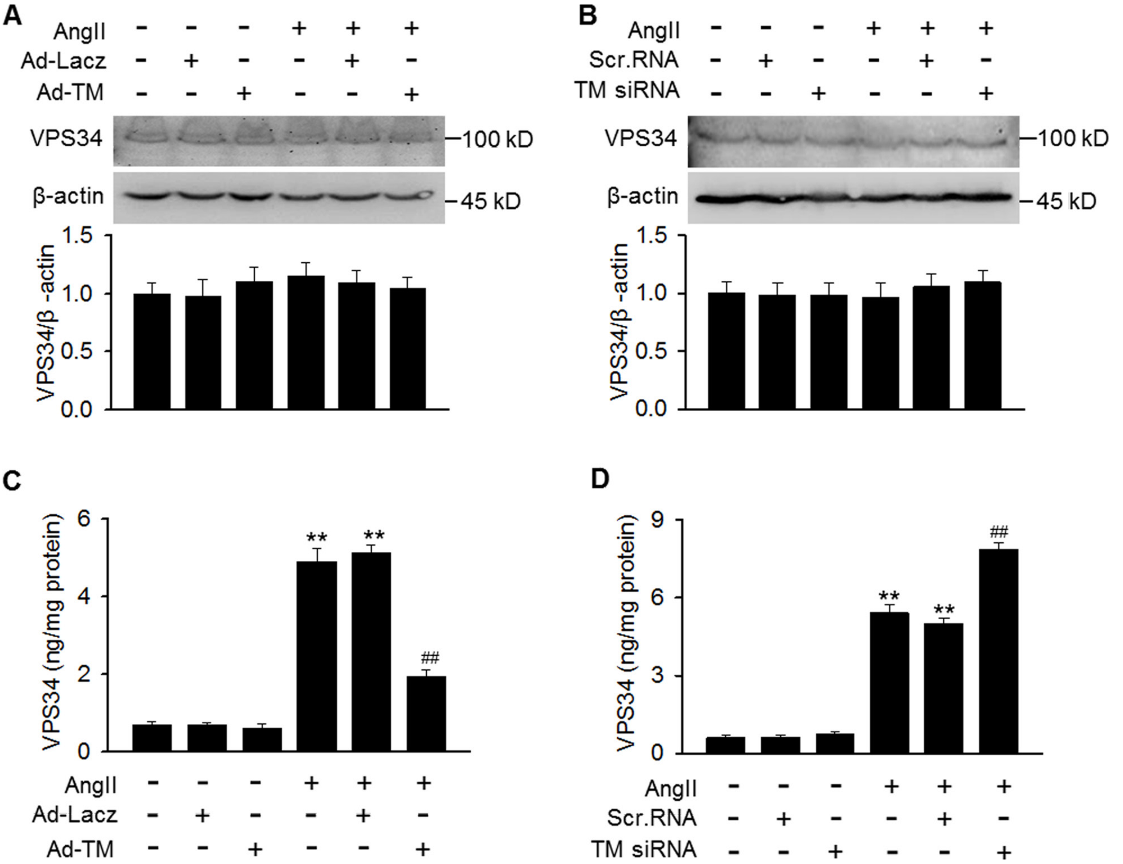

Figure S5. TMEM16A blocked the AngII-induced increase in VPS34 activity in MASMCs. (A–C) The VPS34 expression (A and B) and activity (C and D) in cells transfected with Ad-Lacz or Ad-TM (A and C, respectively), or transfected with scr.RNA or TM siRNA (B and D, respectively) for 48 h before AngII treatment for 24 h. **P < 0.01 vs. control; ##P < 0.01 vs. AngII, one-way ANOVA. n = 5.

Figure S6

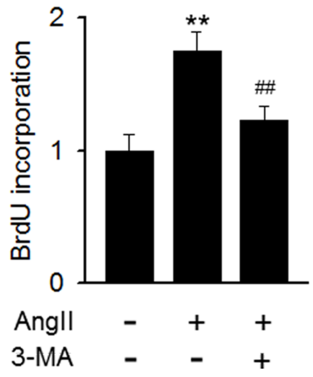

Figure S6. Autophagy inhibition attenuated AngII-induced MASMC proliferation. MASMCs were treated with AngII and 3-MA (5 μmol/L) for 24 h. Cell proliferation was determined by performing BrdU assays. **P < 0.01 vs. control; ##P < 0.01 vs. AngII, one-way ANOVA. n = 6.

Figure S7

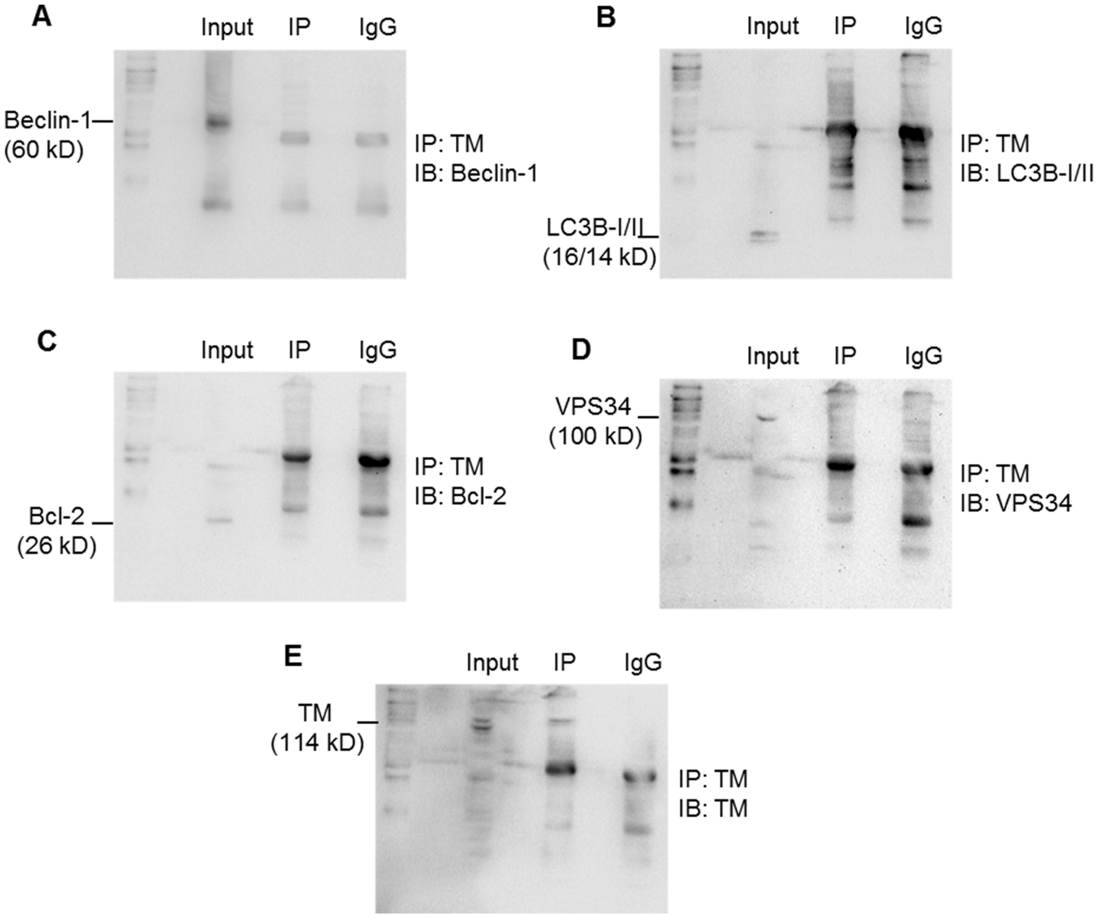

Figure S7. TMEM16A did not bind with Beclin-1, LC3B, Bcl-2, or VPS34 in MASMCs. (A–E) Cell lysates of MASMCs were immunoprecipitated (IP) with an antibody against TMEM16A (TM) and immunoblotted (IB) with antibodies against Beclin-1 (A), LC3B (B), Bcl-2 (C), VPS34 (D), or TMEM16A (E). n = 5.

Figure S8

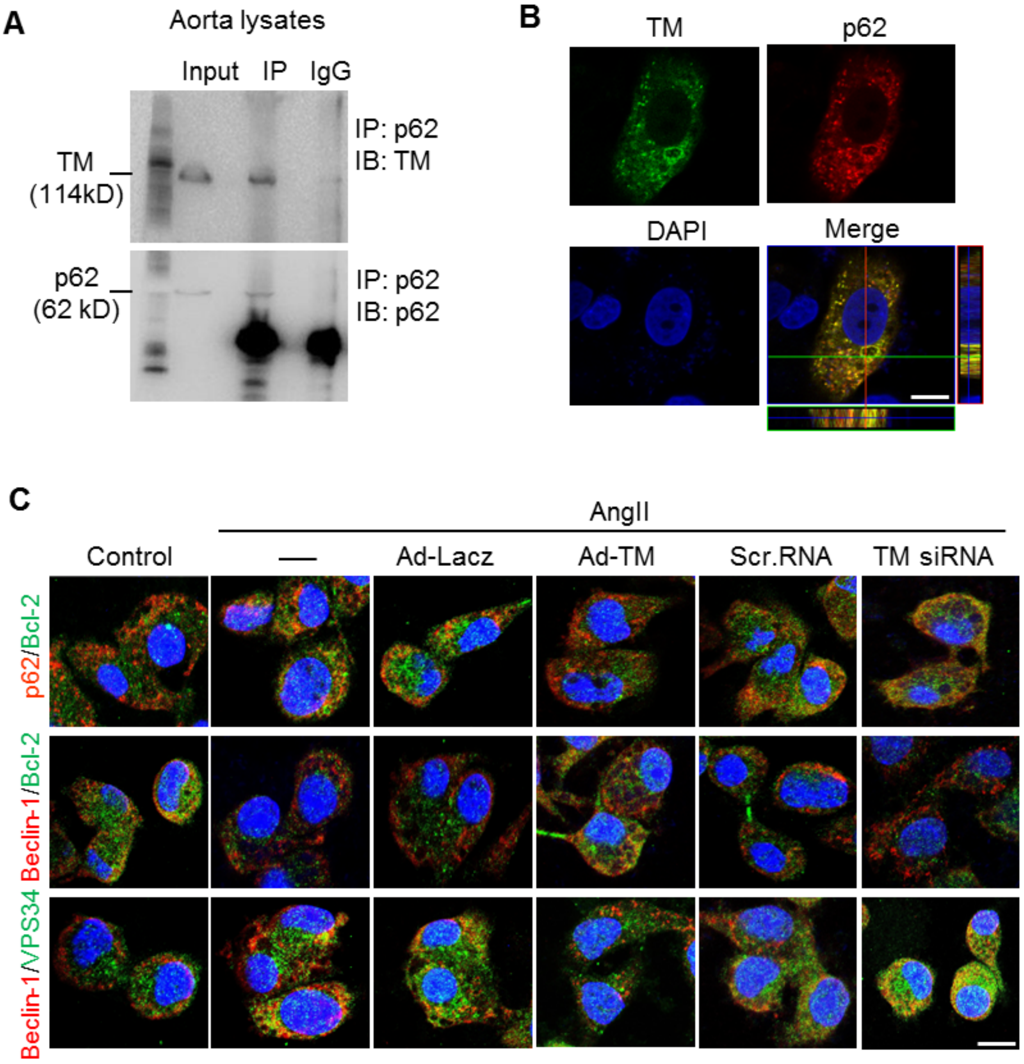

Figure S8. TMEM16A interacted with p62 and regulated p62/Bcl-2/Beclin-1/VPS43 complexes formation. (A) Immunoprecipitation (IP) followed by immunoblotting (IB) in aorta lysates showing presence of TMEM16A in p62 immunoprecipitates. n=5. (B) Representative confocal images of TMEM16A and p62 distribution in normal MASMCs. n=10 cells. (C) MASMCs were treated with Ad-Lacz, TM siRNA or their corresponding negative control for 48 h and then treated with AngII for 24 h. Representative colocalization images of p62/Bcl-2, Bcl-2/Beclin-1 and Beclin-1/VPS34 in MASMCs. n = 100 cells from 5 independent experiments.
